# Supplementary material for: Proteomic traits vary across taxa in a coastal Antarctic phytoplankton bloom
Source: ISME J. 2021 Sep 4;16(2):569–79. doi: 10.1038/s41396-021-01084-9 (PMC8776772; doi:10.1038/s41396-021-01084-9)
Supplement: Supplementary file 2 — Supplemental Material [file 41396_2021_1084_MOESM2_ESM.docx]

# 1 Supplementary Methods

We simulated metaproteomes *in silico* to examine biases arising from inferring taxon-specific proteomes. The primary challenge of inferring taxon-specific coarse-grained proteomes is that not all coarse-grained pools (groups of proteins performing some functional role) are equally identifiable. This also extends to taxa – some taxa are more closely related, and therefore have fewer unique peptides. For example, some coarse-grained pools are easily mapped to a given taxon while others have very few taxon-specific peptides.

To address these expected biases, we created *in silico* metaproteomic datasets (generative model), and sampled the data similar to how a mass spectrometer would (sampling model). We then compared the sampled data to the known dataset and evaluated which conditions biases would arise.

## 1.1 Generative Model

We generate *p* unique peptides, assigned to *k* coarse grained pools, belonging to an organism *j*. We simulate peptides rather than proteins, as peptides are injected into a mass spectrometer with bottom-up mass spectrometry. To simulate different levels of sequence diversity present across protein pools, we generate *k* sequence ‘banks’ of different sizes. Peptide sequences banks are created by randomly sampling from all amino acids, generating a sequence ranging in length from 5–15 amino acids per peptide. An organism-specific peptide profile is created, which randomly samples from each ‘sequence bank’. So a smaller ‘sequence bank’ would represent a coarse grained protein pool with low sequence diversity, and vice-versa.

We then assign abundances to each peptide. Peptide abundance is generated using a random sample from a gamma distribution with the shape parameter of 0.15 and the scale parameter of 10. We chose this distribution as it is similar to the distribution of peptides observed in single-organism proteomics (specifically it has overdispersion, non-zero values only, and is continuous). We then multiply each peptide abundance by a taxonomic abundance unique to each taxon *j*, and by the abundance within a given coarse grained pool *k*. Both the taxonomic abundance and the coarse-grained pool abundance values are similarly drawn from a gamma distribution, except with a shape value of 1. For example, to calculate peptide abundance we first draw a value for an organism abundance (e.g. 100) and multiply that by a value drawn for a coarse-grained pool abundance (e.g. 5). Lastly, we generate a value for all peptides from within this organism and coarse-grained pool (e.g. 2), and multiply these three values. In this case, the intensity of the peptide would be 1000. Once the ‘true’ dataset is generated, we then filter this dataset to create an ‘observed’ dataset, because peptides that are the same from the ‘true’ dataset should be summed. From this observed dataset, we calculate peptide mass and assume a peptide charge state of 2.

## 1.2 Sampling Model

Mass spectrometers sample and fragment peptides for identification, and there is some stochasticity in this sampling process, particularly when using data-dependent acquisition (DDA). Using DDA, peptides are sampled according to their intensity. We subsample our ‘observed dataset’ using a simplistic model of a mass spectrometer. Our model assumes a constant ion peak width, and randomly assigns elution times to peptides from a uniform distribution. A similar version of this model has been extensively validated [48], but the key difference here is including dynamic exclusion and top-N sampling.

We describe sampling model algorithmically below (Algorithm 1). We begin by sorting and then binning elution times for all peptides (steps 1–2). We then loop through every *n*th elution time bin, where *n* represents the number of ions selected for Top *n* DDA (step 4). So with more ions selected for ‘fragmentation’ the mass spectrometer would have less time to scan intact peptides, as is true for instruments that move between scanning MS1 in an Orbitrap and fragmenting peptides in a linear ion trap. Then, if a peptide is on the dynamic exclusion list and it has been on the list for longer than the dynamic exclusion time, it is removed from the dynamic exclusion list (step 4–5). All of the *m/z* windows belonging to a peptide on the dynamic exclusion list are then blocked for sampling (steps 6–7). Of the remaining peptides, we select the top *n* in terms of abundance, and assume that these peptides are identified (step 8). The final step is adding the identified peptides to the dynamic exclusion list (step 9), which prevents those *m/z* regions from being subsequently sampled for a short period of time (the dynamic exclusion time).

*
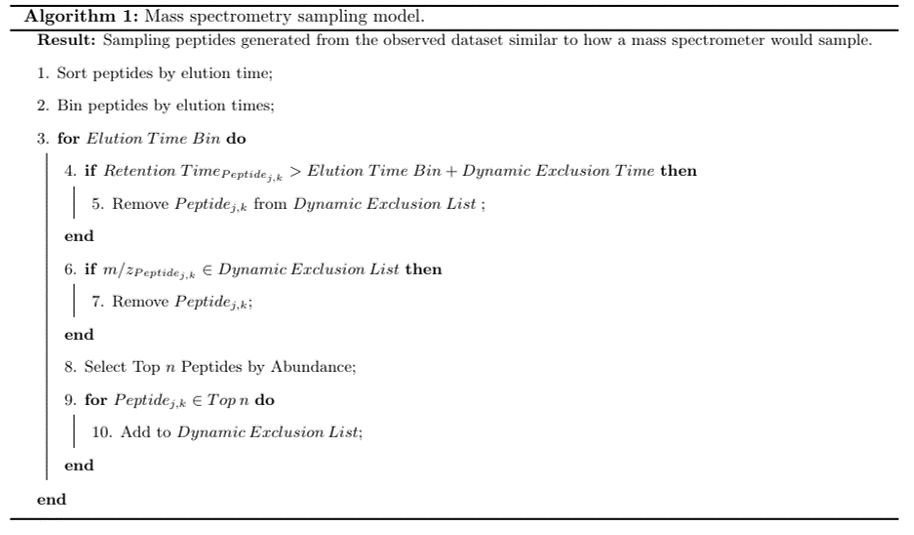
*

## 1.3 Model Parameters

We generated 15 datasets with the following characteristics. Each dataset contained 30 distinct taxa with four coarse-grained protein groups of varying diversity. As above, diversity is modeled using varying sizes of sequence ‘banks’ (we used sizes of 15000, 50000, 100000, 250000, and 500000). From each protein group (represented by these different sequence banks of peptides), each organism has 2000 peptides, which are randomly drawn from these sequence banks.

Retention times are assigned from a uniform distribution ranging from 0–90 minutes.

The maximum injection time, which is used as the width of the elution time bin, is 500ms

(or 0.00833 minutes), following from [48]. We assign a constant ion peak width of 0.5

minutes, independent of ion intensity. We use a Top *n* of 12 ions. Our precursor selection

window is set to 3*m/z* and our dynamic exclusion time span is set to 0.5 minutes.

# 2 Supplementary Discussion

## 2.1 Underestimation of coarse-grained protein groups

Our simulations showed that abundant protein groups have good estimates (close to the 1:1 line, Supplementary Fig. S12), while low abundance protein groups tend to be underestimated. Each point in Supplementary Fig. S12 represents an estimate of taxon-specific coarse-grained protein pool, with the “true” value compared with the “observed” value.. This relationship of underestimation with abundance is because of the data-dependent acquisition sampling method that mass spectrometers use. Data-dependent acquisition specifically targets the highly abundant peptides, so lower abundance groups tend to get sampled less. The method we (and others) typically use is to sum the peptide intensities to obtain an abundance estimate. With fewer peptides quantified, the sum will be lower (Supplementary Fig. S12). Note that sequence diversity can also influence these estimates (represented with blue colour gradient, Supplementary Fig. S12), but only until there is extremely low diversity (darkest colour), corresponding with only a few peptides identified and mapped to a taxon. Note that we are considering the proteomic mass fraction, and quantified this using peptide intensities. Protein quantification typically adjusts for the length per protein, but if this adjustment was not made, it would be equivalent to how we are calculating the proteomic mass fraction.

## 2.2 Sequence Diversity

Our conclusions about the ribosomal and photosynthetic proteomic mass fractions, as well as the environment-independent proteomic mass fraction, are potentially influenced by varying degrees of biodiversity within each taxonomic group. Yet, we restricted our analyses to these taxonomic groups due to the robustness of estimation with higher numbers of peptides (above simulations, and [48]). Further, this level of taxonomic resolution is typically used to compare ecological strategies across marine microbes, so we reasoned it would be useful to introduce these proteomic traits at the same level (e.g. [22]).

Here we outline the challenges in comparing taxonomic groups with varying biodiversity within each group, focusing on the environment-independent mass fraction proteomic ‘trait’. Biodiversity could influence the environment-independent mass fraction in several ways, depending on the exact meaning of ‘biodiversity’ in this context. The source of this variation could be due comparisons between taxonomic groups with varying levels of biodiversity (in terms of sequence diversity), or it could be due to a shift in community composition within taxonomic groups across samples (for example from one diatom species to another). These different mechanisms lead to different potential problems. For example, if community composition is constant across time, but one grouping is more biodiverse than another, our estimates could be interpreted as an average across subgroups (note this is not necessarily the case). But if there are significant shifts in community composition, then this might correspond with an apparent increase in peptide variability that arises from the change in community composition rather than changes in protein expression.

How could varying degrees of diversity be adjusted for? Simply correcting for total peptide diversity (in terms of numbers of peptides unique for a taxonomic group) is an obvious first step. Consider, however, the relationship between the total number of unique peptides for a species with a high regulatory cost (many regulatory proteins). There would be a causal connection between the number of unique peptides and the exact trait we are examining – regulatory cost – so ‘adjusting’ for peptide diversity would not be appropriate.

Another approach to assess variable biodiversity across taxa is to examine finer taxonomic resolution, and then estimate and compare the environment-independent proteomic mass fraction at that finer resolution with our original, coarse resolution estimates. This is problematic for two reasons: 1) peptides used for a finer taxonomic resolution are unlikely to be a random subsample, and certain protein functions are most likely enriched. If these protein functions are more or less likely to be constitutively expressed, estimates will not be comparable across taxonomic resolution would not be comparable. 2) Subsampling in mass spectrometry is explicitly biased towards highly abundant peptides. Peptides that are more abundant tend to have lower coefficients of variation (Supplementary Fig. S15). So, a subsample will systematically bias the environment-independent mass fraction upwards. We have outlined some of the principal challenges associated with using metaproteomics to estimate this proteomic trait, and future work is needed to address these issues. However, we think that this trait is still worth examining, because it likely underpins key aspects of ecological variability (e.g. as examined theoretically and experimentally in *E. coli*; [59]).

## 2.3 Abundance-Noise Relationship

Another potential bias in studying the environment-independent protein mass fraction is that less abundant proteins have more variation across identical conditions, as the mean protein coefficient of variation is negatively correlated with mean protein abundance in cultures (Supplementary Fig. S15; [51]). So, identifying more peptides would increase the average coefficient of variation. However, we did not observe a negative correlation between the peptide-specific coefficient of variation and the mean peptide abundance (Supplementary Fig. S16), suggesting that this bias does not influence our estimated environment-independent peptide mass fraction.

**Supplementary Table Caption**

Supplementary Table 1. Sequencing and assembly characteristics for the three assemblies (one metagenomic and two metatranscriptomic) used for databases of potential proteins for searching mass spectra. Sheet 1 corresponds to the metagenomic and metatranscriptomic sequencing conducted on the GOS-927, GOS-930, GOS-933 and GOS-935 filters (see Methods). Sheet 2 corresponds to the metatranscriptomic experiment described in the Methods.

**Supplementary Figures**

Figure S1. Representation of the overlap between different database configurations and the number of tryptic peptides within each. Bar graphs on top represent the number of peptides identified with a given set of sequence groups (i.e. overlapping databases). The set of overlapping sequence groups is represented below with points and lines. For example, the first column shows that the metatranscriptome experiment (all samples) contained by far the greatest number of tryptic peptides, and that the metatranscriptome experiment (T=0) had no unique tryptic peptides (because it is a subset of the former). The side bar plot, next to the database configuration name, is the total number of peptides within each sequence group database (note the scale of the numbers are between 0 and 1$\times$10^7^). In this figure, only the smallest filter size is shown (0.1 μm).


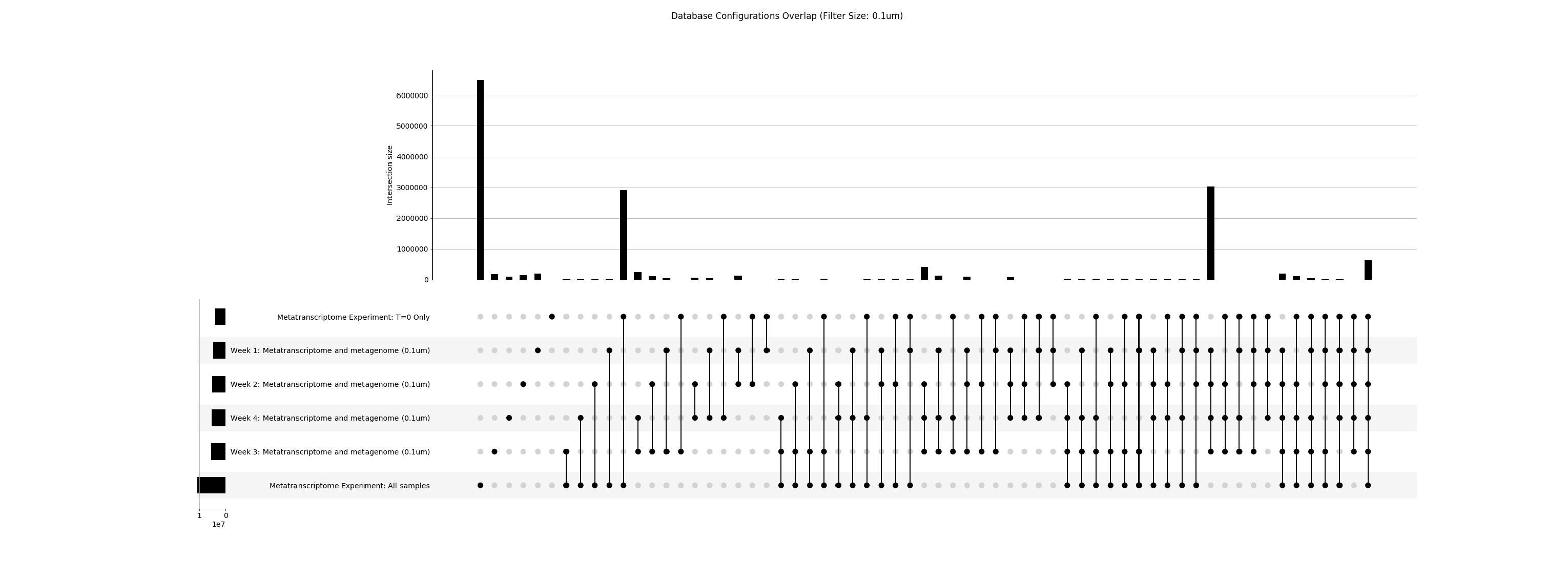


24

Figure S2. Representation of the overlap between different database configurations and the number of tryptic peptides within each. Bar graphs on top represent the number of peptides identified with a given set of sequence groups (i.e. overlapping databases). The set of overlapping sequence groups is represented below with points and lines. The side bar plot, next to the database configuration name, is the total number of peptides within each sequence group database (note the scale of the numbers are between 0 and 1$\times$10^7^). In this figure, only the medium filter size is shown (0.8 μm).


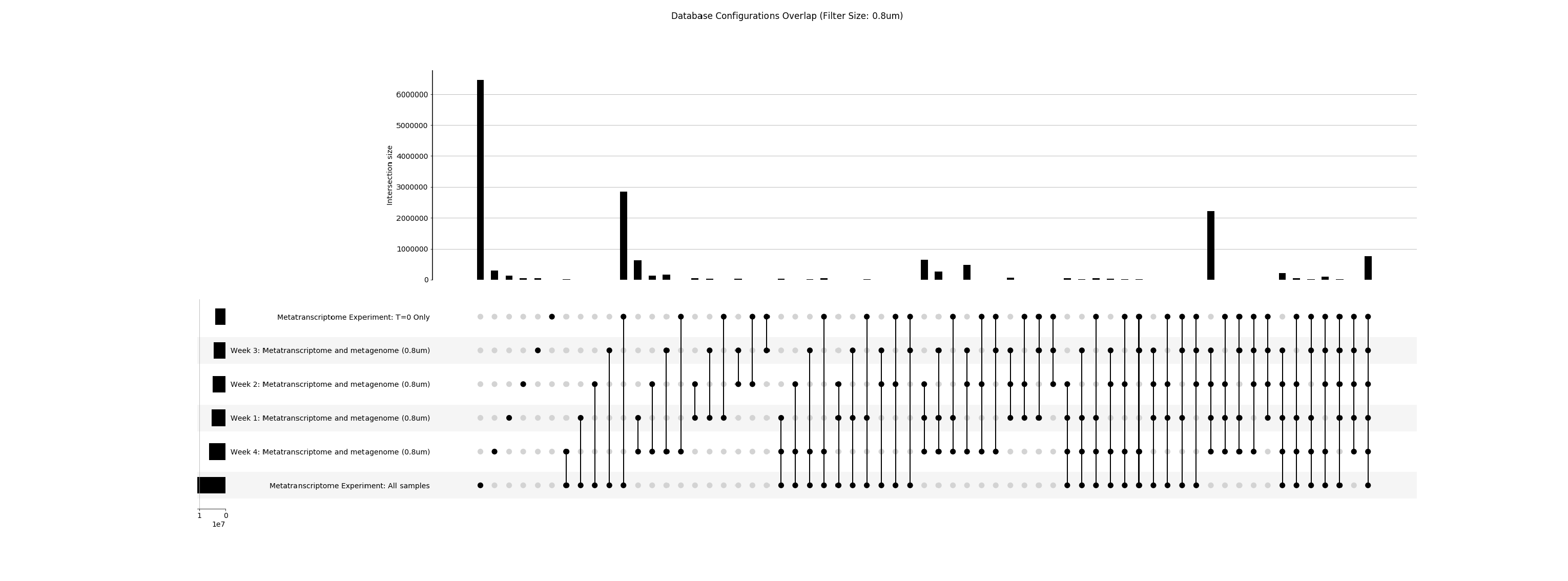


25

Figure S3. Representation of the overlap between different database configurations and the number of tryptic peptides within each. Bar graphs on top represent the number of peptides identified with a given set of sequence groups (i.e. overlapping databases). The set of overlapping sequence groups is represented below with points and lines. The side bar plot, next to the database configuration name, is the total number of peptides within each sequence group database (note the scale of the numbers are between 0 and 1$\times$10^7^). In this figure, only the largest filter size is shown (3.0 μm).


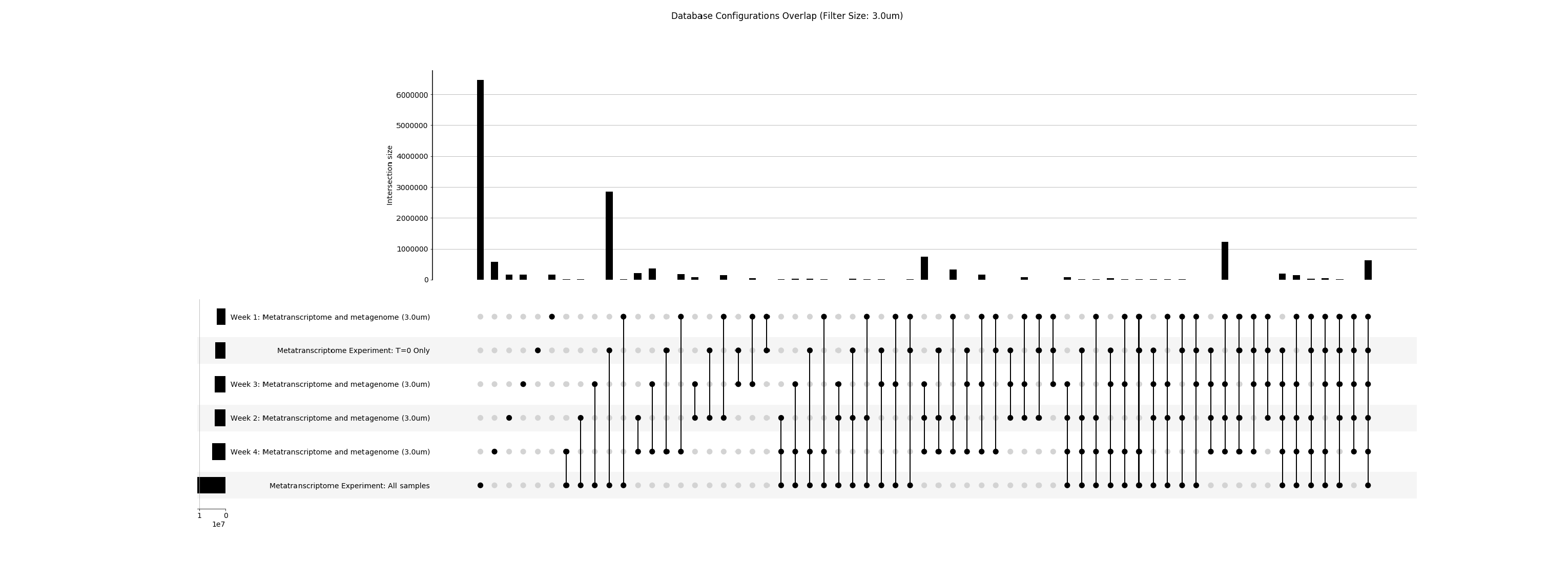


26


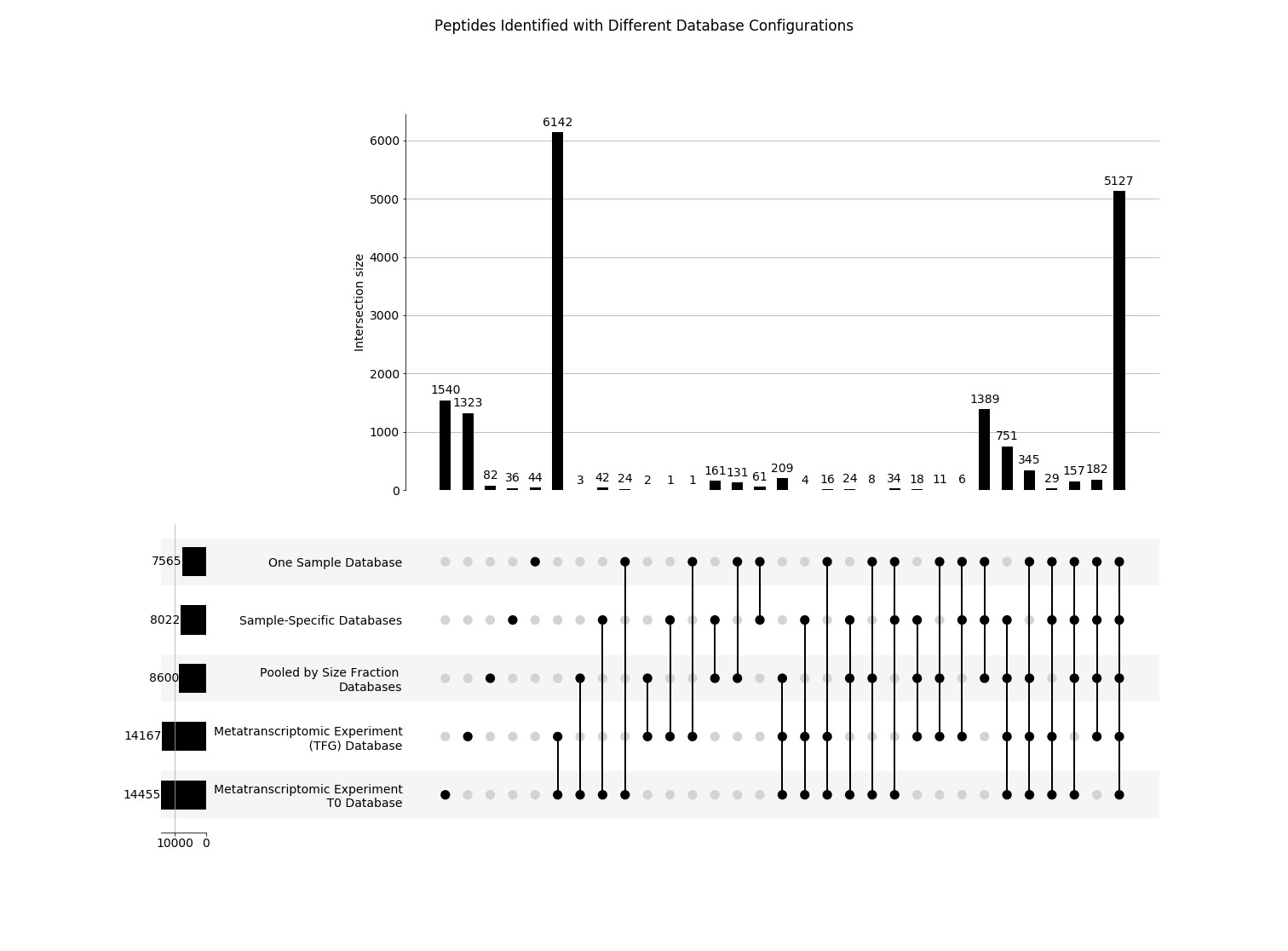


Figure S4: Representation of the overlap between different database configurations and the number of peptides identified with each. Bar graphs on top (with numbers above) represent the number of peptides identified with a given set of databases (i.e. overlapping databases). The set of overlapping databases is represented below with points and lines. For example, the first column on the left represents peptides uniquely identified using the database ‘Metatranscriptome Experiment T0’, where 1540 peptides were uniquely identified. The side bar plot, next to the database configuration name, is the total number of peptides identified using each database. In this figure, all filter sizes are summed together.


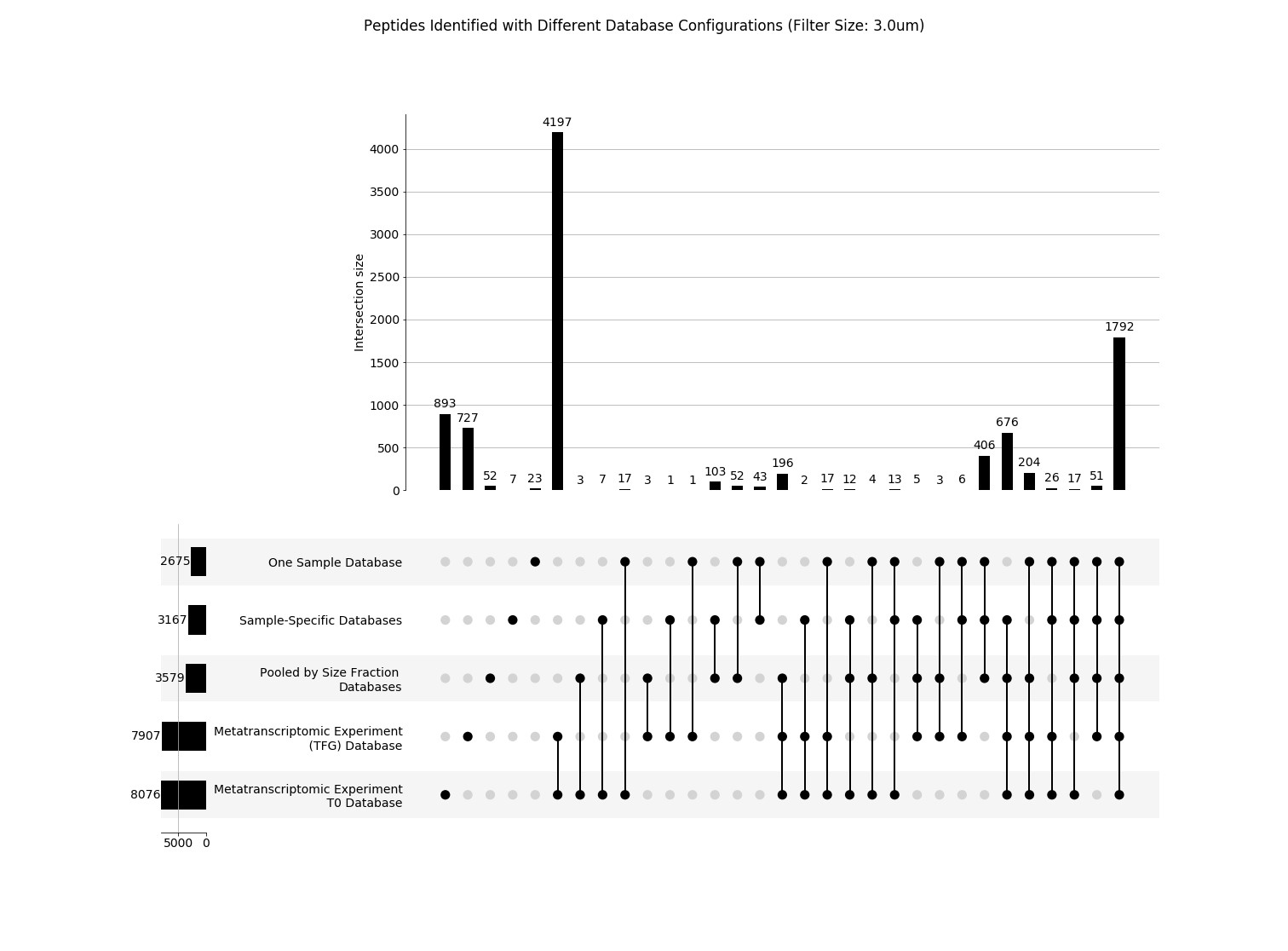


Figure S5: Representation of the overlap between different database configurations and the number of peptides identified with each. Bar graphs on top (with numbers above) represent the number of peptides identified with a given set of databases (i.e. overlapping databases). The set of overlapping databases is represented below with points and lines. The side bar plot represents the total number of peptides identified using each database. In this figure, only the largest filter size is shown (3.0 *µ*m).


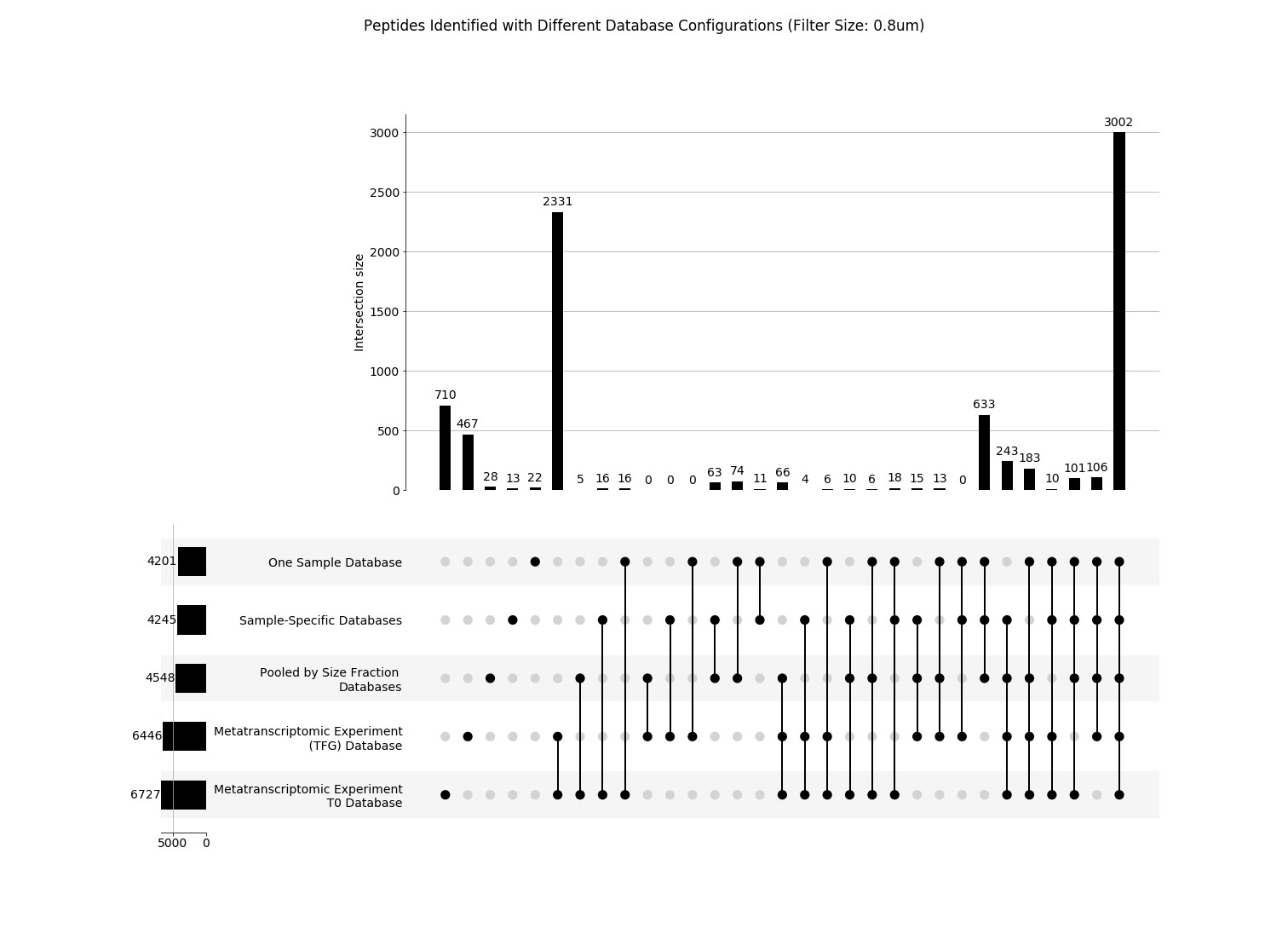


Figure S6: Representation of the overlap between different database configurations and the number of peptides identified with each. Bar graphs on top (with numbers above) represent the number of peptides identified with a given set of databases (i.e. overlapping databases). The set of overlapping databases is represented below with points and lines. The side bar plot represents the total number of peptides identified using each database. In this figure, only the middle filter size is shown (0.8 *µ*m).


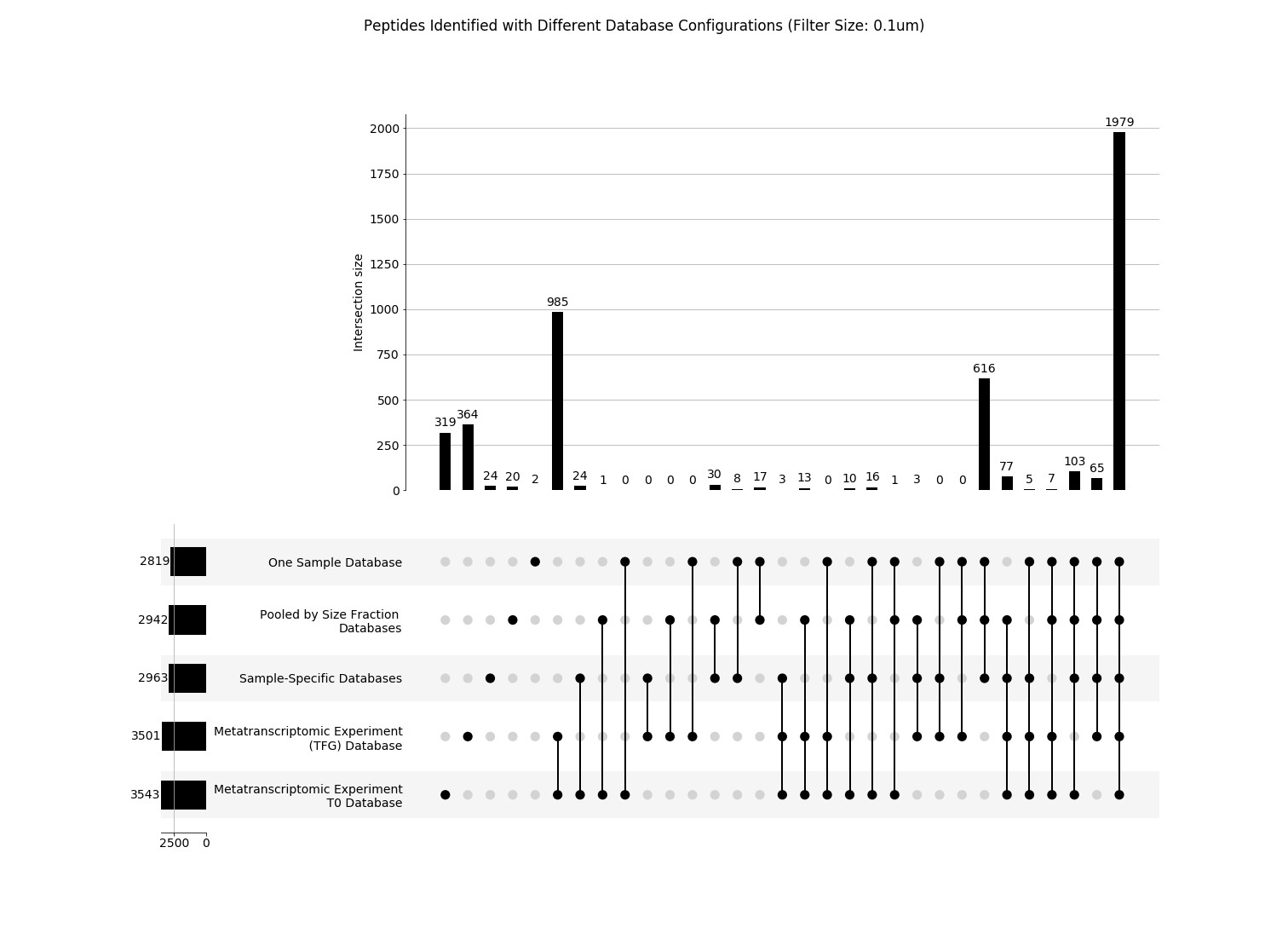


Figure S7: Representation of the overlap between different database configurations and the number of peptides identified with each. Bar graphs on top (with numbers above) represent the number of peptides identified with a given set of databases (i.e. overlapping databases). The set of overlapping databases is represented below with points and lines. The side bar plot represents the total number of peptides identified using each database. In this figure, only the smallest filter size is shown (0.1 *µ*m).


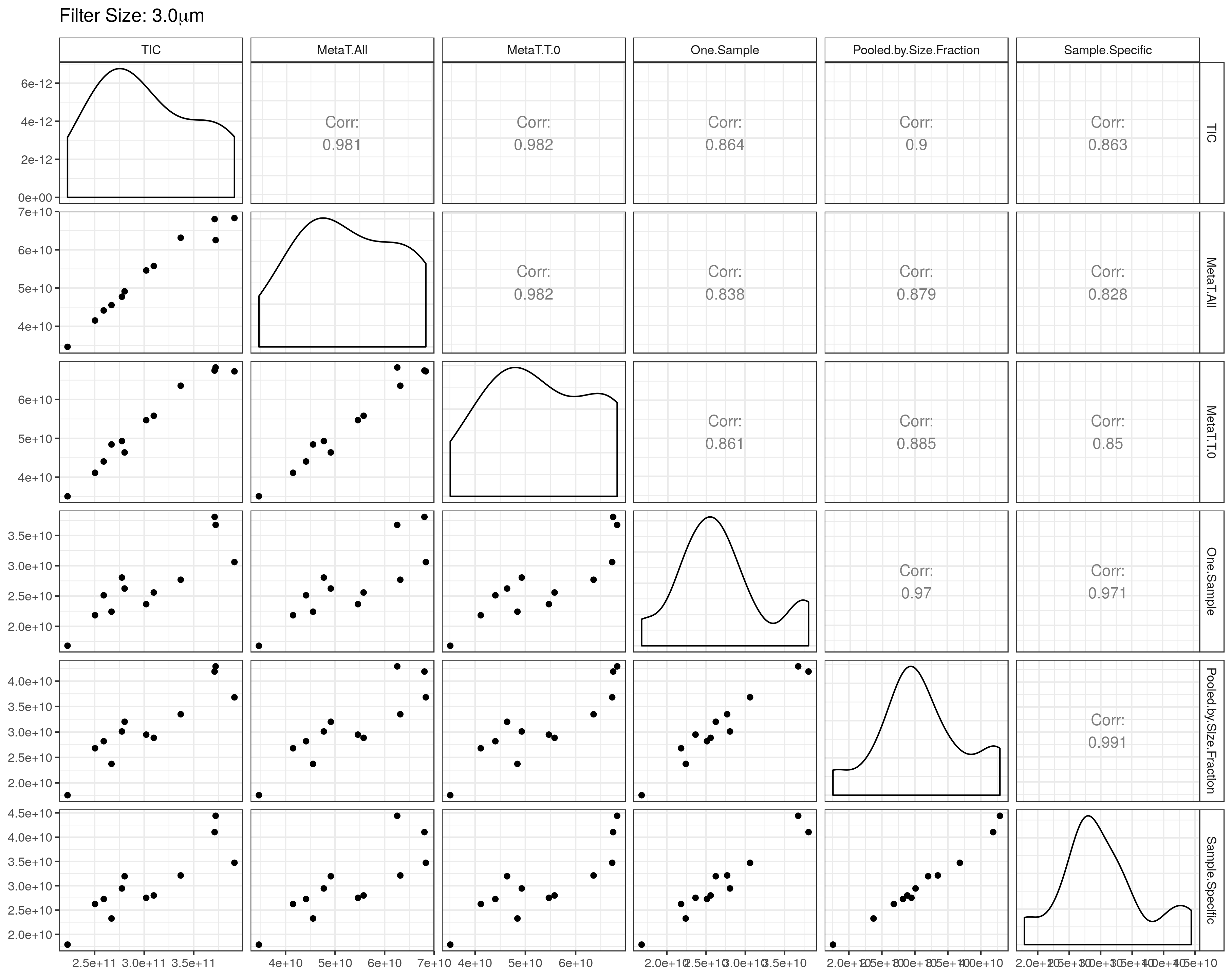


Figure S8: The sum of peptide intensities (i.e. normalization factors) are against each other for different database configurations, as well as against total ion current (TIC). Points represent different mass spectrometry experiments. Correlation values (coefficient of determination) are represented in corresponding locations. Only the largest filter size is shown here (3.0 *µ*m).


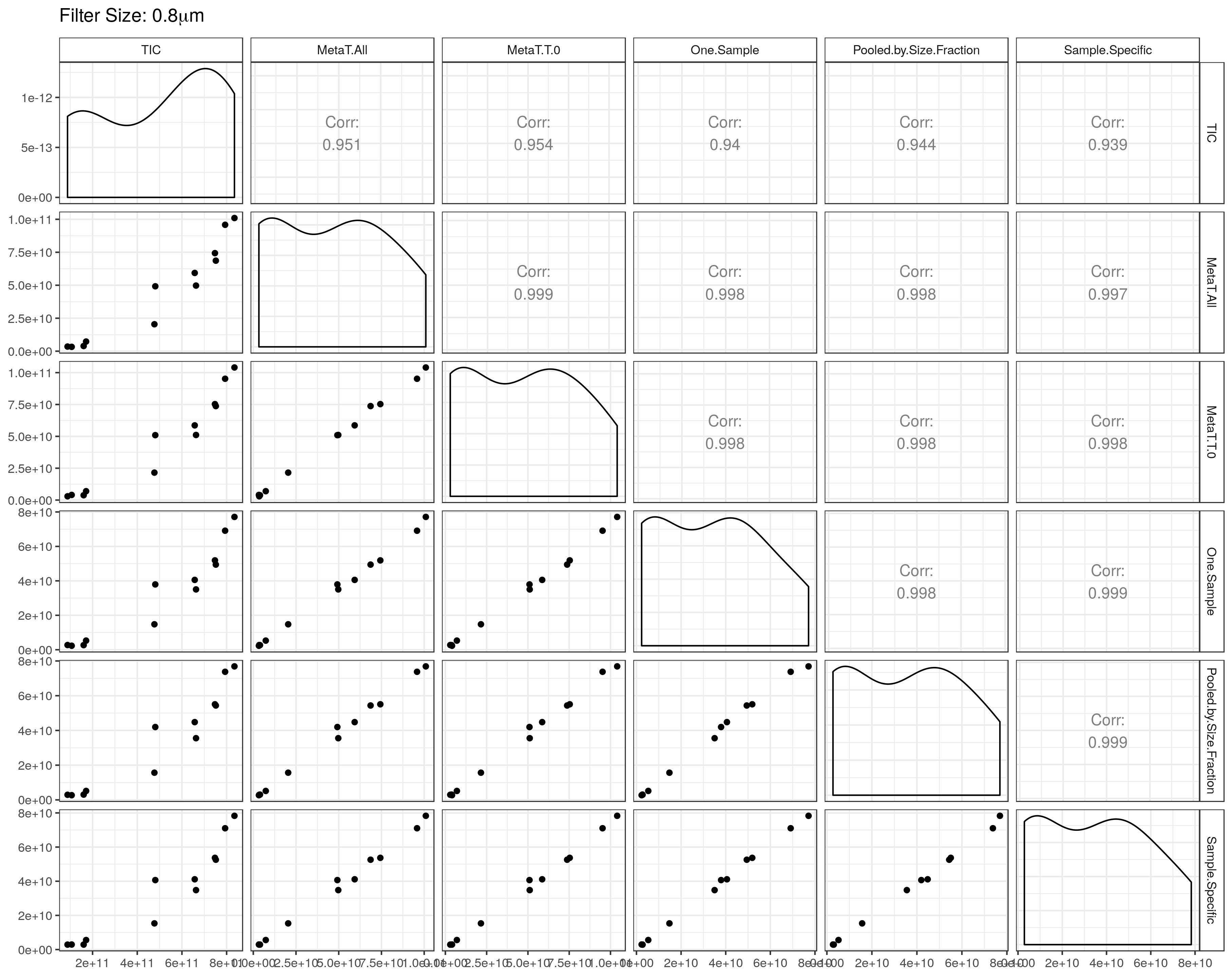


Figure S9: The sum of peptide intensities (i.e. normalization factors) are against each other for different database configurations, as well as against total ion current (TIC). Points represent different mass spectrometry experiments. Correlation values (coefficient of determination) are represented in corresponding locations. Only the middle filter size is represented here (0.8 *µ*m).


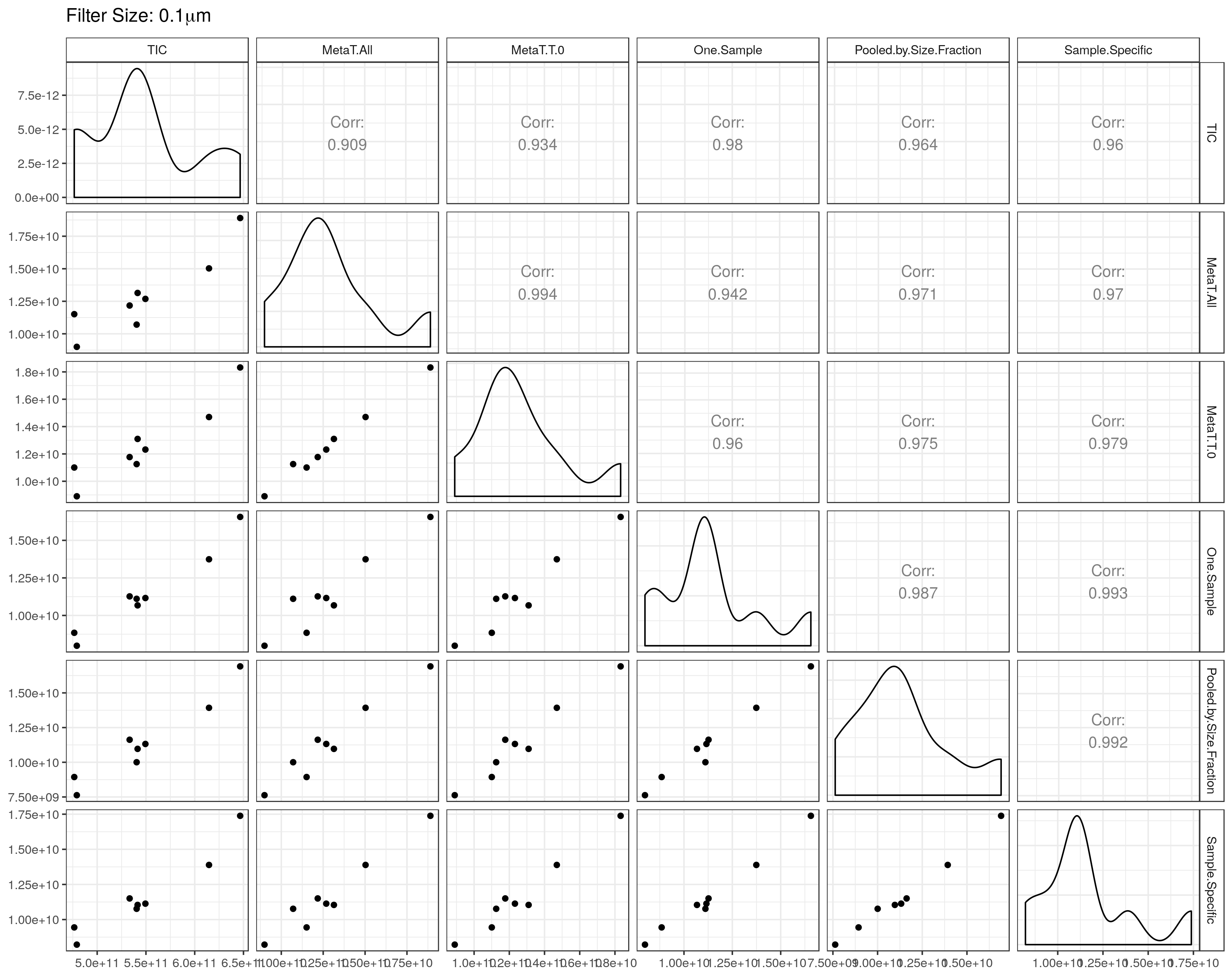


Figure S10: The sum of peptide intensities (i.e. normalization factors) are against each other for different database configurations, as well as against total ion current (TIC). Points represent different mass spectrometry experiments. Correlation values (coefficient of determination) are represented in corresponding locations. Only the smallest filter size is represented here (0.1 *µ*m).


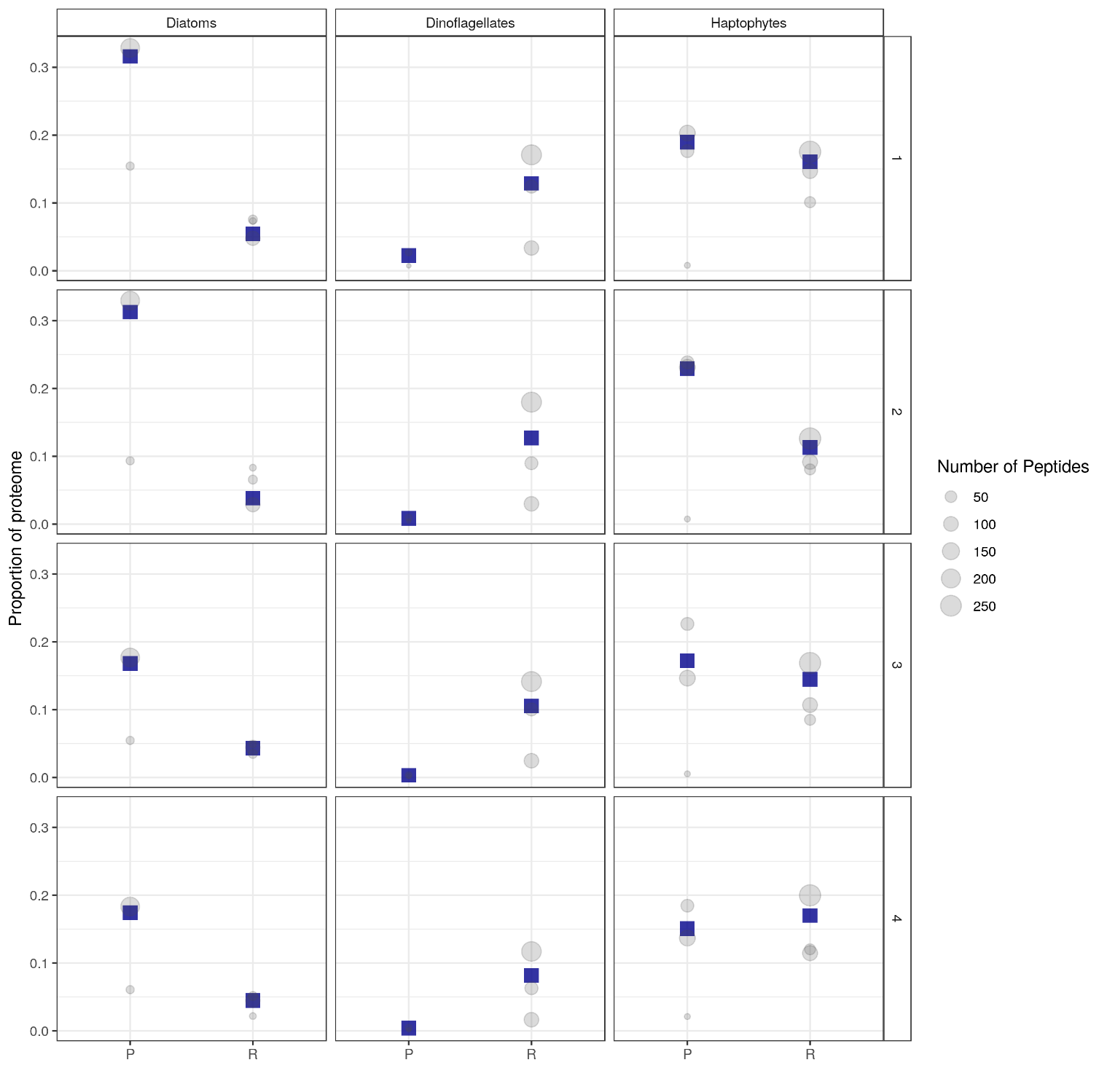


Figure S11: Demonstrating how various estimates across filters are collapsed into one estimate, based on the number of peptides identified within each filter size. Grey points represent the different filter sizes, while the size per grey point is the number of peptides observed in that filter, corresponding to a given taxa or a coarse-grained proteomic pool (P is photosynthetic protein pool, R is ribosomal protein pool). Dark blue squares are the weighted estimates. Numbers in the vertical direction (right side) correspond to the different sampling weeks.


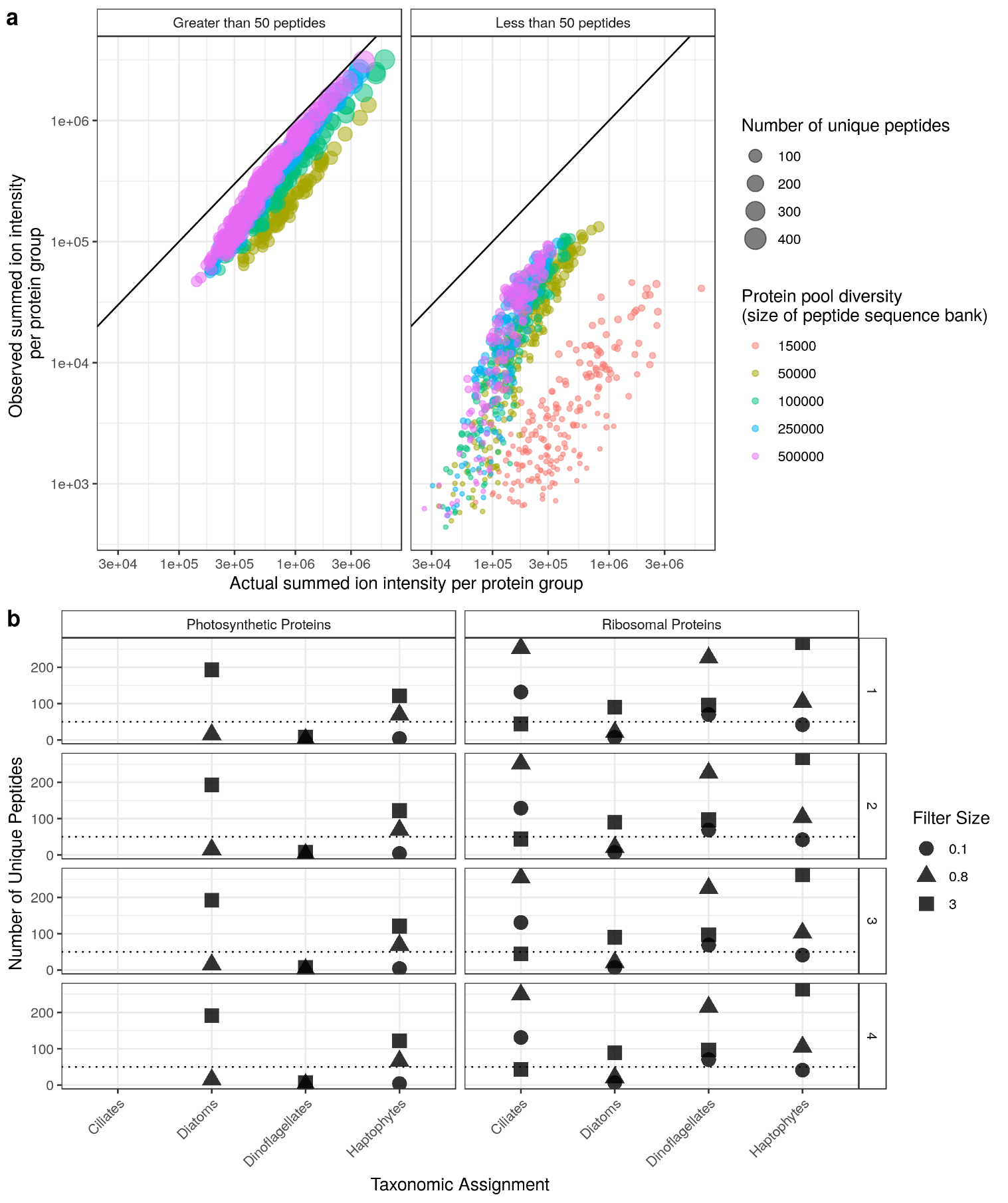


Figure S12: Results from the metaproteomic sampling simulation suggest that restricting analyses to protein groups and taxa that are abundant will prevent bias due to different amounts of diversity. **a**, varying degrees of simulated diversity (colour of points), demonstrates that low diversity pools are underestimated (i.e. far away from 1:1 line). Yet, if many peptides are observed (i.e. above 50 peptides), then the estimates are linearly correlated with the 1:1 line, with only slight underestimates due to diversity. **b**, the number of photosynthetic and ribosomal protein specific peptides that are also taxon-specific across different filter sizes (estimates across filter sizes were weighted and merged). At least one filter for each protein pool has greater than 50 peptides, except Dinoflagellate photosynthetic proteins. The dotted horizontal line corresponds with 50 unique peptides. These data suggest that our estimates of ribosomal and photosynthetic protein mass fraction not susceptible to diversity-induced bias.


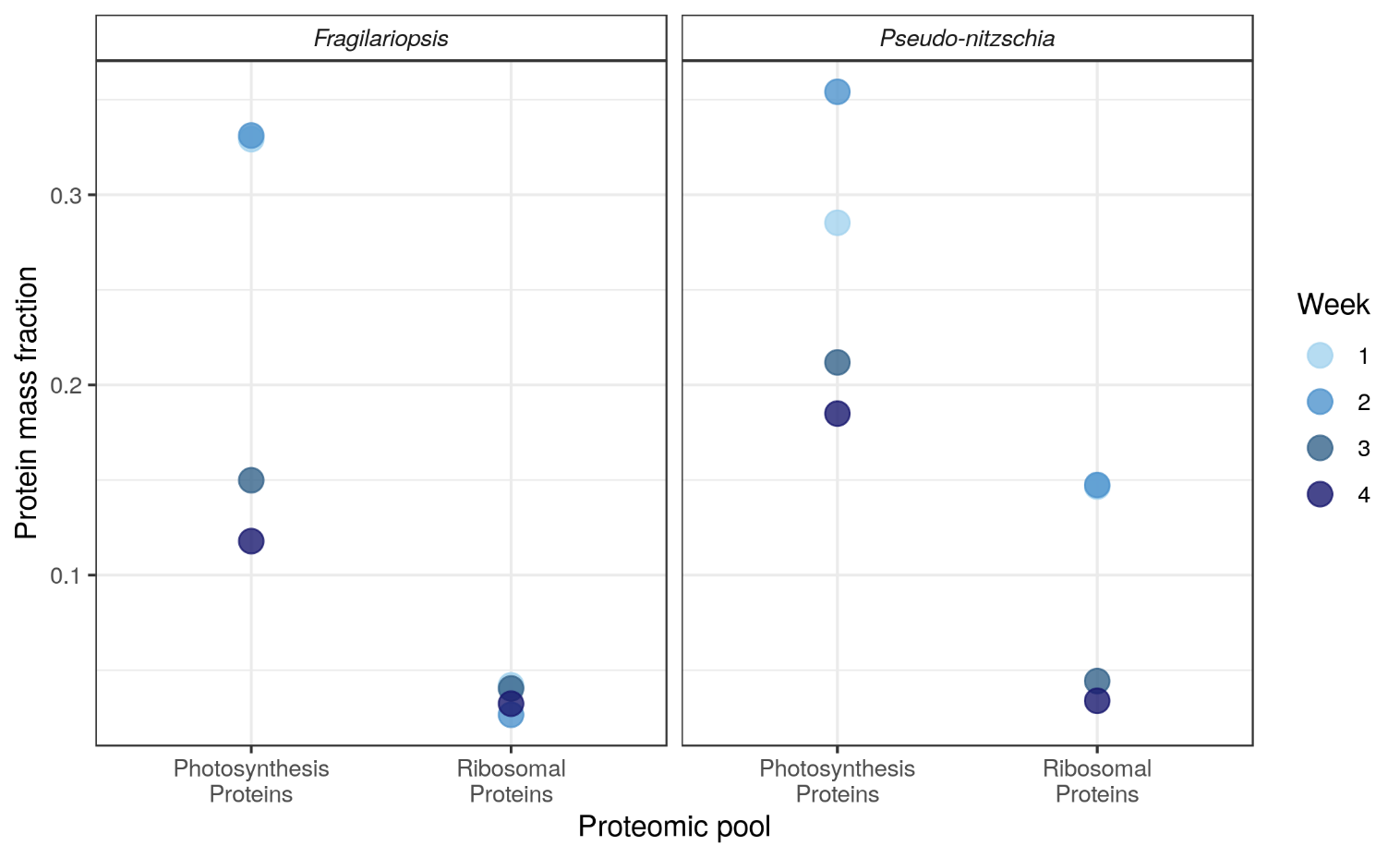


Figure S13: Proteomic proportions of two taxonomic groups of diatoms, *Fragilariopsis* sp*.* and *Pseudo-nitzschia* sp. Ribosomal and photosynthetic proportions were similar across groupings, and also similar to the larger grouping of diatoms.


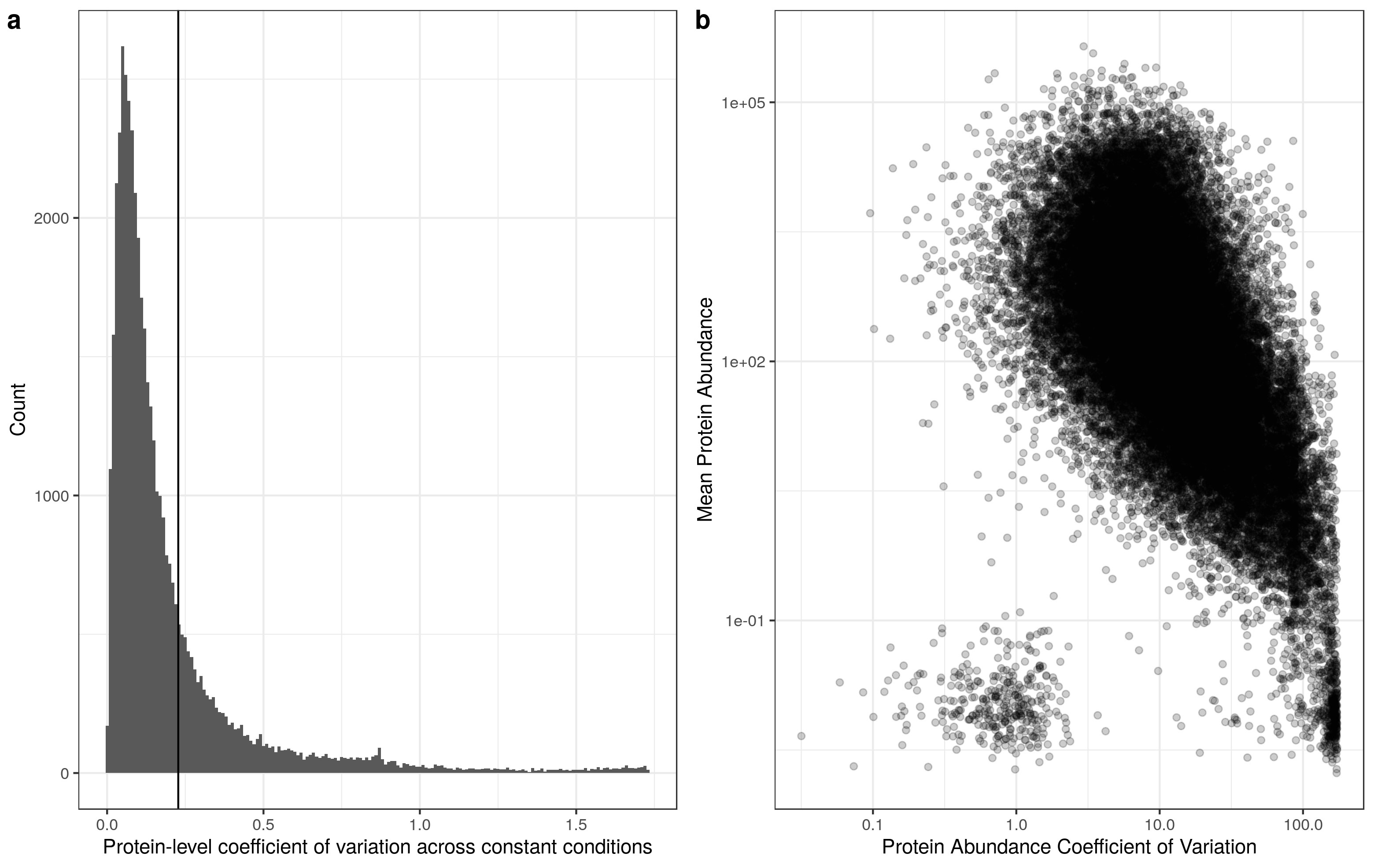


Figure S14: Protein-level summary statistics derived from a comprehensive proteomic characterization of *E. coli* [51]. **a.** the distribution of protein-level coefficients of variation, using all 22 experimental treatments described in [51]. The vertical line was calculated by first determining the third quartile of the distribution of coefficients of variation for each condition, and then calculating the mean of these third quartiles. These coefficients of variation should presumably lead to mostly constant protein expression, but there are some proteins that have intrinsic noise in expression levels. We chose an arbitrary cut-off to classify protein expression as constant or not. **b.** Plotting the relationship between the mean protein abundance across conditions with its’ coefficient of variation shows a negative correlation between the two summary statistics at the protein level (Spearman’s ρ = -0.55).


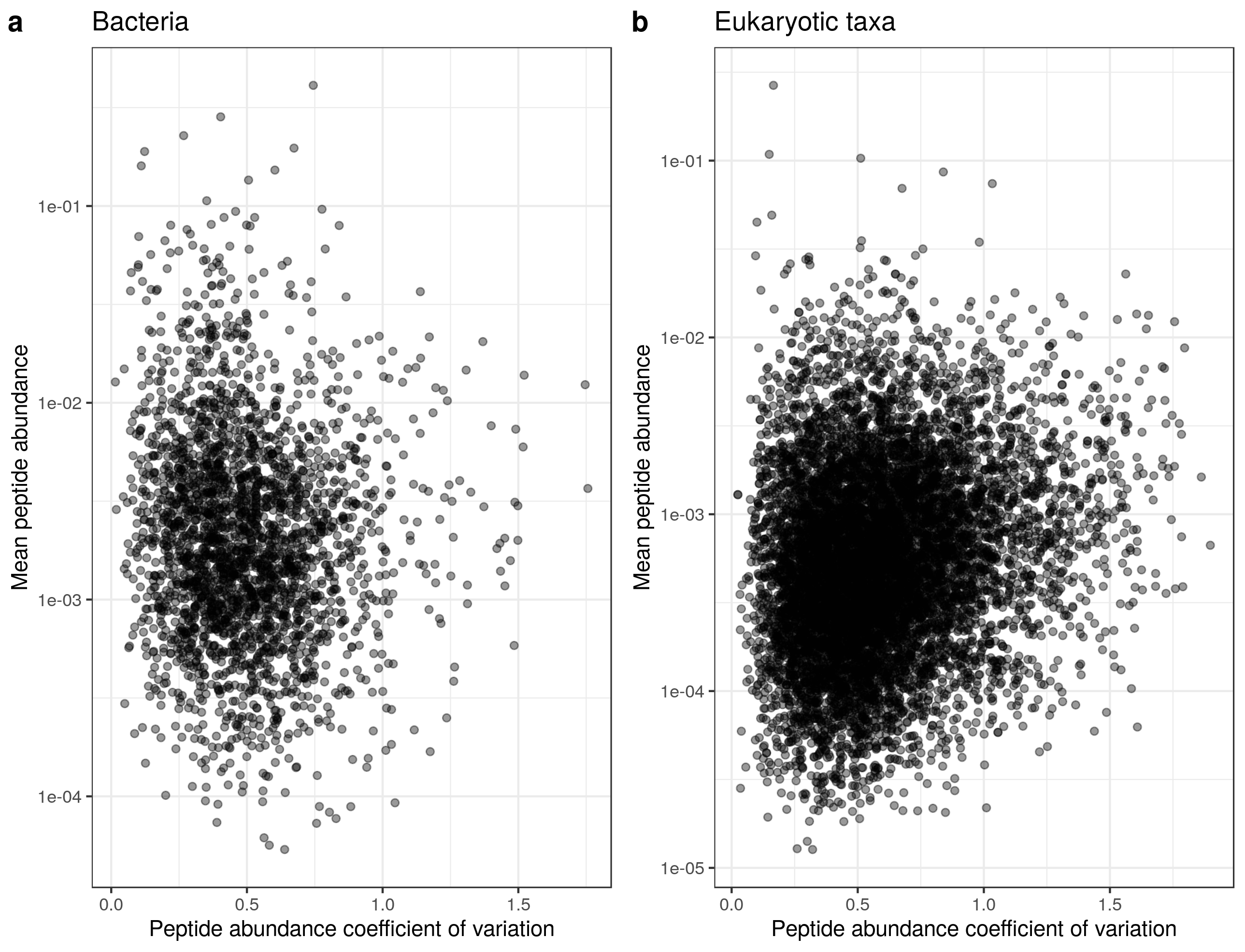


Figure S15: Weak relationships between the peptide abundance coefficient of variation and the mean peptide abundance for the prokaryotic and eukaryotic taxa we observed (Spearman’s ρ = -0.09 and 0.18, respectively).


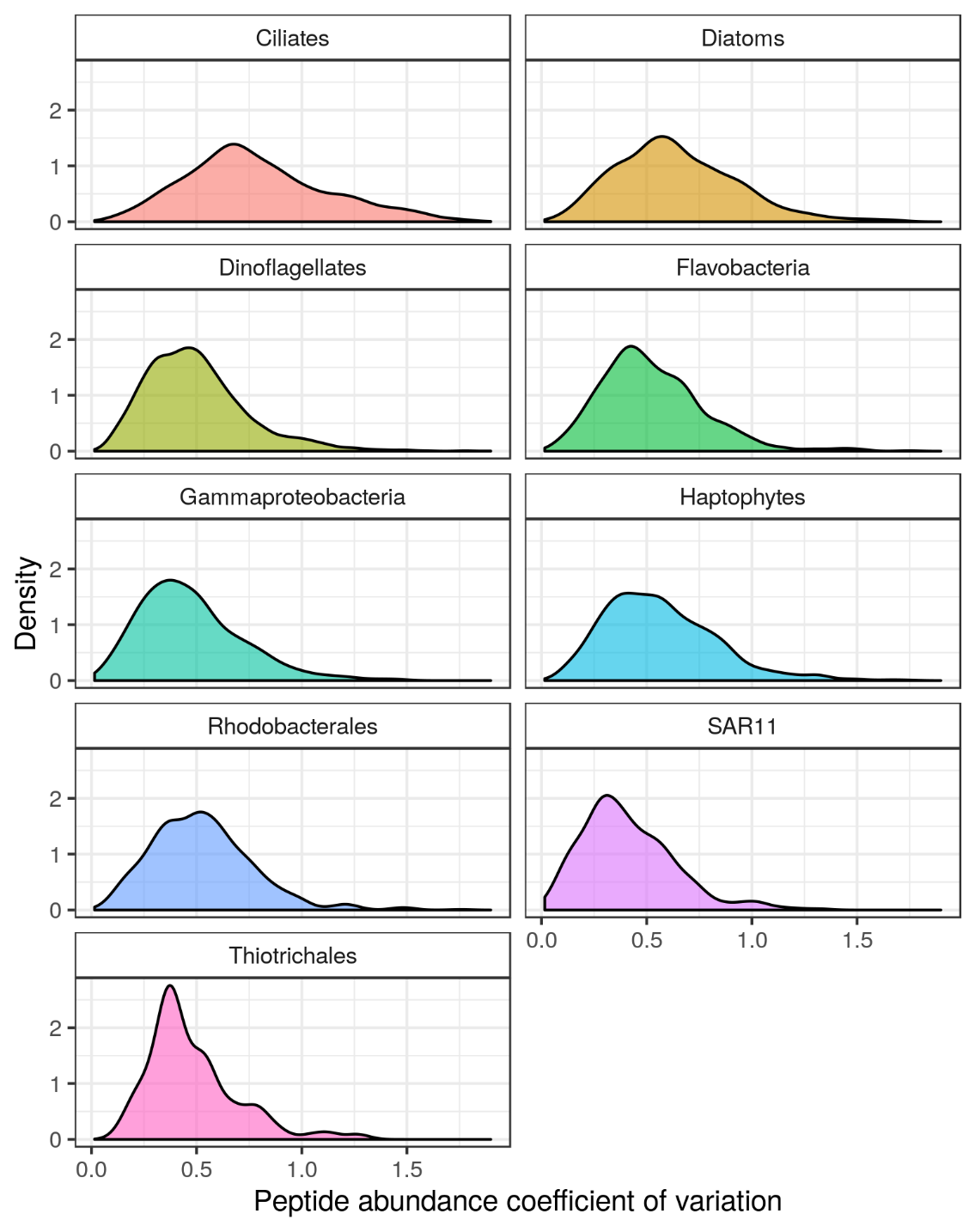


Figure S16: Distributions of peptide-specific coefficients of variation for each taxa we examined. In the main manuscript, only SAR11 and diatom distributions are shown. Methods for calculating this distribution are given in the main manuscript.
